# The association between pediatric chronic pain clinic attendance and health care utilization: A retrospective analysis

**DOI:** 10.1080/24740527.2017.1415701

**Published:** 2018-01-30

**Authors:** Fiona Campbell, Jennifer Stinson, Carley Ouellette, Vitali Ostapets, Garry Salisbury

**Affiliations:** a The Department of Anesthesia and Pain Medicine, The Hospital for Sick Children, Toronto, ON, Canada; b The Department of Anesthesia, University of Toronto, Toronto, ON, Canada; c Child Health Evaluative Sciences, The Hospital for Sick Children, Toronto, ON, Canada; d Lawrence S. Boomberg Faculty of Nursing, University of Toronto, Toronto, ON, Canada; e Health Services Branch, Negotiations and Accountability Management Division Ministry of Health and Long Term Care, Kingston, ON, Canada

**Keywords:** health care utilization, chronic pain, chronic pain clinic, pain management, pediatrics

## Abstract

**Background:**

Pediatric chronic pain is common, disabling, and costly. Children with chronic pain have high health care utilization in that they are seen by multiple health care providers, have frequent emergency room visits, and require many diagnostic tests. Pediatric health care utilization relating to direct health care services and associated costs of attendance at chronic pain clinics in Canada has not been well described.

**Aim:**

The purpose of this project was to analyze the cost of physician services for individuals attending an interprofessional pediatric chronic pain clinic over an 8-year span including years before, during, and after treatment.

**Methods:**

Physician claims were extracted from the Ontario Health Insurance Plan (OHIP) Claims History Database and retrospectively reviewed over 8 fiscal years for 100 new patients seen at the Chronic Pain Clinic at the Hospital for Sick Children. The utilization metrics analyzed included physician consultations and follow-up appointments, emergency room visits, lab tests, and diagnostic imaging. These data reflected 2 years prior to the first chronic pain clinic appointment, year of initial appointment, and five subsequent years.

**Results:**

Health care utilization based on OHIP claims related to physician services and cost analysis showed an increase during the 2 years prior to first chronic pain clinic appointment, a decrease during the year of initial appointment, and a further decrease over the subsequent 5 years.

**Conclusions:**

Further prospective research is required to establish whether attendance at the chronic pain clinic caused the reduction in health care services and costs and, if so, to identify the effective components of treatment.

## RÉSUMÉ

**Contexte**: La douleur chronique pédiatrique est fréquente, invalidante et coûteuse. Les enfants qui souffrent de douleur chronique sont de grands utilisateurs des services de santé car ils sont suivis par de multiples prestataires de soins, se rendent souvent à l’urgence et nécessitent de nombreux tests diagnostiques. L’utilisation de services de santé pédiatriques en ce qui concerne les services de santé directs et les coûts afférents à la fréquentation de cliniques de la douleur chronique au Canada est peu documentée.

**But**: Ce projet avait pour but d’analyser le coût des services médicaux pour les personnes fréquentant une clinique interprofessionnelle de la douleur chronique pédiatrique pendant une période de huit ans couvrant les années avant, pendant et après le traitement.

**Méthodes**: Les demandes de paiement des médecins ont été extraites de la base de données portant sur l’historique des demandes de paiement auprès de l’Assurance-santé de l’Ontario (OHIP) et ont été examinées rétrospectivement sur une période de huit exercices financiers pour 100 nouveaux patients suivis à la clinique de la douleur chronique de l’Hôpital pour enfants malades. Parmi les mesures relatives à l’utilisation qui ont été analysées, on compte : les consultations auprès de médecins et les rendez-vous de suivi; les visites à l’urgence; les tests de laboratoire; et l’imagerie diagnostique. Les données portaient sur les deux années précédant le premier rendez-vous à la clinique de la douleur chronique, l’année du premier rendez-vous et les cinq années suivantes.

**Résultats**: En se fondant sur les demandes de paiement présentées à l’OHIP, l’analyse de l’utilisation et des coûts des services de santé a démontré que ces derniers avaient augmenté au cours des deux années précédent le premier rendez-vous à la Clinique de la douleur chronique, qu’ils avaient diminué pendant l’année du premier rendez-vous et qu’ils avaient à nouveau diminué au cours des cinq années suivantes.

**Conclusions**: D’autres études prospectives sont nécessaires pour établir si la fréquentation d’une clinique de la douleur chronique a donné lieu à la réduction de l’utilisation et des coûts des services de santé et, le cas échéant, pour cerner les composantes du traitement qui sont efficaces.

## Introduction

Chronic pain is a common condition affecting more than 6 million Canadians of all ages and negatively impacts health-related quality of life.^1^^–4^ Although chronic pain is more common in the middle-aged and elderly,^4^ studies have reported a high prevalence and negative impact in older children and adolescents.^2–5^ Chronic pain, defined as pain that persists beyond expected healing time or recurrent pain occurring at least three times over a period of 3 months, includes varying levels of disability.^6–8^ A recent systematic review of population-based studies found a high prevalence of chronic pain in adolescents, with rates from 8% to 40%, of whom 5% have severe pain-related disability.^8^

Early pain management intervention can reduce pain duration, pain-related disability, and thus health care expenditures.^9–11^ However, recent surveys have revealed unacceptably high levels of undertreatment and intolerably long wait times (often >1 year) to access appropriate chronic pain care in Canada.^11,12^ Moreover, in 2007, Peng et al. reported the status of dedicated multidisciplinary pediatric chronic pain clinics in Canada and found only five, leaving many provinces and regions without access to subspecialty pediatric chronic pain care.^13^ In 2013, an unpublished updated review concluded that this number had increased to eight^14^ and, since then, the number of Canadian pediatric chronic pain clinics has only increased to ten. Moreover, some provinces and rural areas in Canada are still without access to pediatric pain clinics.

Given the negative impact of unrelieved or undertreated pain on health-related quality of life^15–17^ and physical and psychosocial functioning, specialized interprofessional chronic pain teams are now considered the optimal therapeutic standard of care for individuals with chronic pain conditions.^17,18^ Evaluation by an interprofessional chronic pain team leads to a tailored comprehensive pain management plan for children and adolescents, which encourages improved function, promotes self-management, and ultimately results in less reliance on multiple health providers and health care cost reduction.^18–23^

The Ontario Ministry of Health and Long Term Care (MOHLTC) established a Pediatric Chronic Pain Network to address this important health issue, one of the objectives of which was to improve access to interprofessional pediatric chronic pain clinics in Ontario.^24^ One of the overarching mandates of the network is to evaluate the impact of attendance at interprofessional pediatric chronic pain clinics on health care utilization. The purpose of this study was to analyze the trajectory of direct health care services and associated costs based on physician claims to the Ontario Health Insurance Plan (OHIP) related to attendance at a pediatric chronic pain clinic.

## Methods

### Design

A retrospective design was used to review OHIP claims over 8 fiscal years for 100 new referral patients seen at the Chronic Pain Clinic at the Hospital for Sick Children (SickKids), Toronto. The 100 patients were randomly selected out of a total of 106 eligible new admissions in 2009. These data reflected 2 years prior to first chronic pain clinic appointment (fiscal year [FY] 2007–2008), year of initial appointment (January 1, 2009, to December 31, 2009; FY 2009), and five subsequent years (FY 2010–2014). OHIP claims were reviewed by the MOHLTC to extract health care utilization data relative to all physician services and included number of emergency room visits, physician consultations and follow-up appointments, overall physician services, and the average cost of physician services per patient per fiscal year. The cost is estimated based on approved amount instead of paid amount because some physicians participate in alternative payment programs and submit shadow-billed claims paid at zero instead of fee-for-service claims. This project was approved by the Department of Quality and Risk Management at the Hospital for Sick Children as a quality improvement project, therefore permitting data examination without review by the Research Ethics Board.

#### Setting

During the study period, the clinical interprofessional chronic pain team at SickKids was composed of anesthesiologists, advanced practice nurses, physiotherapists, psychologists, and a psychiatrist. This team collectively meets with patients and families in person and/or via telemedicine in an outpatient-based clinical setting and provides care for children and adolescents up to 18 years of age.

#### Inclusion and exclusion criteria

Inclusion criteria included all fee-for-service and shadow-billed claims submitted by Ontario physicians to OHIP for patients enrolled into chronic pain clinic for the period of 8 fiscal years 2007–2014. Exclusion criteria included (1) duplicates (explanatory code: 32,35,36), (2) Workplace Safety Insurance Board claims.

## Results

### Demographic data

One hundred OHIP numbers were submitted for review, of which six were excluded as follows: four because the patients died of causes unrelated to chronic pain and did not have data spanning the study period, one because it was an invalid OHIP number, and one because it was an outlier with billings indicative of treatments not pertaining to chronic pain. From the remaining sample, 72% (*n* = 68) were female and 28% (*n* = 26) were male. The age ranged from 4.8 to 17.6 years, with a mean age of 13.9 years (SD = 2.93). All patients were diagnosed with chronic pain and were patients of the chronic pain clinic at SickKids.

### Health care utilization—Analysis by physician services

**Figure 1.**
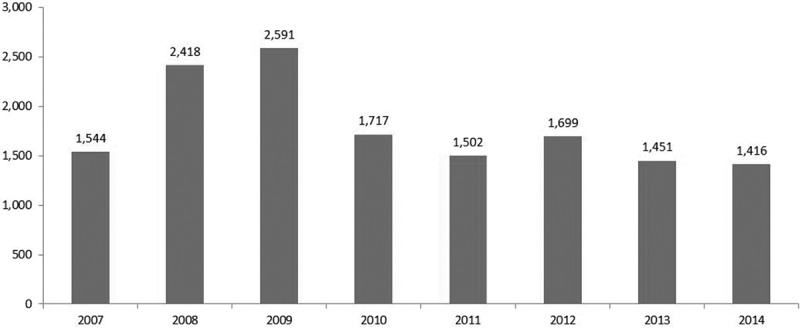
Total number of visits to physicians.

**Figure 2.**
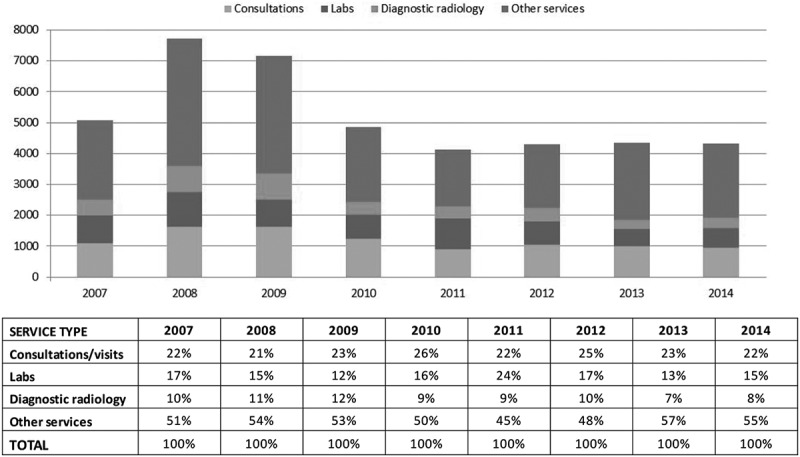
Physician services by type and fiscal year.

Physician services increased by 68% between FY 2007 and FY 2009 until all patients were enrolled in the chronic pain clinic by the end of December 2009. In 2010, the number of physician visits decreased from 2,591 in FY 2009 to 1,717 in FY 2010 (*P* < .0001; Figure 1). During the three subsequent years, the number of physician visits declined further to 1,416 in FY 2014. On average, each patient had been seen by nine physicians in FY 2007, by 13 physicians in FY 2009, and by eight physicians in FY 2014. Physician services were also decreased, with a 40% reduction of services from FY 2009 to FY 2014, which included consultations/visits, labs, diagnostic radiology, and other services (other services include surgical services and diagnostic and therapeutic services; e.g., colonoscopy, angioplasty, echocardiography, ultrasound; Figure 2). Additionally, there was a 36% reduction in visits to the emergency room from 67 in FY 2009 to 43 in FY 2014.

### Health care utilization—Analysis by cost

**Figure 3.**
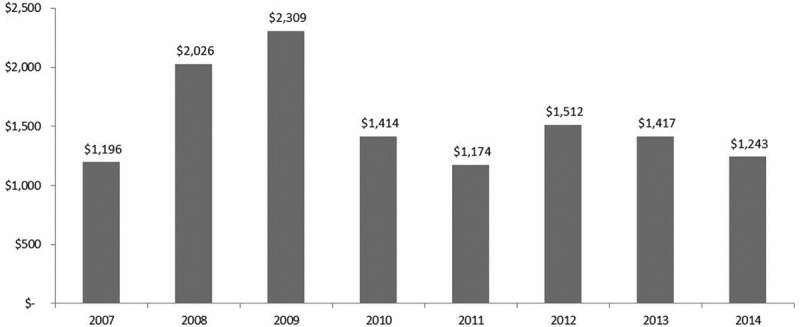
Average cost of physician services per patient by fiscal year.

The MOHLTC excluded one patient number because it was an extreme outlier with billings indicative of treatments not pertaining to chronic pain. The annual cost per patient was estimated based on approved amounts on OHIP claims. Its trend over 8 fiscal years was similar to the trend observed for the total number of visits to physicians (Figures 1 and 3). The maximum cost per patient was $2,591 in FY 2009. It declined to $1,414 in FY 2010 and stayed relatively stable over the following 4 years. Paired *t* test showed that the difference in cost per patient between FY 2014 and FY 2009 was statistically significant (*t* = −4.48, *P* < .0001, degrees of freedom = 90) with a mean difference of $894 (95% confidence limit $498, $1290). OHIP claims data analysis indicated that health care utilization and associated costs relative to all physician services declined for patients under the care of the interprofessional chronic pain clinic.

## Discussion

To our knowledge, this is the first study examining health care utilization associated with attendance at a Canadian pediatric interprofessional chronic pain clinic. We found that health care utilization based on OHIP claims for physician services increased during the 2 years prior to first pediatric chronic pain clinic appointment, decreased during the year of initial appointment, and decreased further over the subsequent 5 years. These costs were related to physician services provided for any reason in any setting, inclding inpatient, outpatient, and community/physician office. Other costs related to hospital-based services and medications were not included because hospital-related costs are not at sufficient detail to link to a specific patient intervention and medication costs are either borne by the patient or private insurance.

Previous studies have shown that health care utilization is high for adolescents with chronic pain.^25,26^ Recently, Groenewald et al. identified economic costs of chronic pain in a cohort of treatment-seeking adolescents receiving interdisciplinary pain treatment through a comprehensive cost of illness study.^25^ They examined a population of 149 adolescents (aged 11–17) diagnosed with chronic pain and found that their cohort of patients had an annual associated cost of $11,787 per participant with a median cost of $ 6,770, of which the largest proportion of reviewed health care utilization costs (68%) was toward direct medical services. These data were representative of pain-related care, which included physician consultations, diagnostic testing, and emergency department visits.^25^ Interestingly, the medical services in this study were much more expensive than in ours, and though the intent of our study was to use physician services as a measure of treatment intensity, it would be interesting for future studies to explore jurisdictional differences in cost.

Similar conclusions were drawn by Hogan et al.^26^ through a retrospective cohort study. In their study, Ontario health care databases and the electronically linked Canadian Community Health Survey were reviewed to determine health care costs and health care utilization related to chronic pain.^26^ Hogan et al. identified adults and adolescents over the age of 12 years and closely matched them to individuals without pain using propensity score matching methods.^26^ They then linked data to determine mean 1-year per person health care costs and health care utilization for each group and mean incremental cost for chronic pain. They found that patients with chronic pain had increased physician visits, emergency room visits, and numbers of hospitalization in comparison to matched controls. The authors note that one of the strengths of their study was that their data may help justify new programs and the authors emphasized that implementation of such programs may be economically advantageous, particularly because chronic pain accounts for approximately 5% of all public health care spending in Canada.^26^

Ample evidence supports that intensive interprofessional pain management programs are the most optimal therapeutic paradigm for patients with chronic pain,^13,18,21^ and our findings in this retrospective analysis suggest that the implementation of more widely accessible pediatric interprofessional chronic pain programs could not only provide patients with the most optimal therapeutic paradigm but also serve as a strategy to address high health care utilization. For example, in a longitudinal observational study of adolescents who received intensive interdisciplinary pain treatment, there were significant findings related to clinical improvement domains (pain intensity, pain-related disability, and pain-related school/work absence) and statistically significant economic effects (admission rate and subjective financial burden).^27^ The authors concluded that intensive interdisciplinary pain treatment is clinically effective and reduced health care utilization over time.^27^ Similar to our conclusions, additional research is required to further identify different moderators and mediators to tailor individualized treatments to achieve the most effective clinical outcomes. Unfortunately, there are few designated pediatric chronic pain clinics within Canada, which consequently leaves pediatric patients waitlisted, untreated, and with suboptimal quality of life.^13,20^ Access to specialized pediatric interprofessional chronic pain clinics is recommended in order to potentially reduce health care costs and improve health outcomes of children and adolescents suffering from chronic pain conditions.^28^ Our findings in part led to the Ontario MOHLTC’s decision to support and significantly expand pediatric chronic pain services across the province.^24^

### Limitations of the study

Despite our important findings, there are weaknesses that limit the conclusions that may be drawn from this analysis. This retrospective analysis did not include a comparator group of patients with chronic pain who were not seen at an interprofessional pediatric chronic pain clinic, so we are unable to comment on the extent to which chronic pain clinic attendance caused the reduction in health care utilization. Our sample size was relatively small, which may limit generalizability of our findings. We were unable to include the small portion of payment information (approximately 5%–7%) stored outside of the claims history database in the cost estimates. Complexity of the physician non-fee-for-service payment model makes cost calculation labor intensive and in some cases impossible for low-granularity data; for example, services or visits. Therefore, an approved amount was used as a proxy for shadow-billed claims, though we know that this slightly underestimates the real cost. In addition, numerous changes to fees in the schedule of benefits make it difficult to normalize the cost estimate in current-year dollars. Lastly, other associated health care utilization costs (e.g., physiotherapy, occupational therapy, or other allied health) could not be captured through this analysis, because these expenditures are not covered through OHIP.

## Unanswered questions, future directions, and conclusions

This retrospective survey demonstrates a reduction in health care utilization for patients attending an interprofessional chronic pain clinic, and though there was no comparator group, these findings suggest that there may be economic benefit provided by these clinics. We are unable to comment on which components of the program were associated with the reduction in health care utilization. Prospective data are required not only to evaluate the impact of attendance at such clinics on health care utilization but also to tease out which treatments or combinations of treatments are most effective for children with chronic pain and their families.^29–31^

Chronic pain programs are expensive and in the current climate of escalating health costs, it would be helpful to contrast costs with cost savings. Unfortunately, lack of other treatment costs available through OHIP makes this very difficult to address. However, having shown that there can be a reduction of service provision intensity for children who are managed in a chronic pain clinic, there may be value in exploring a more detailed analysis of cause and effect as well as deeper financial analysis. It is important for policymakers to have good information to make choices where to provide investments in services.

Future directions include the proposed development of a provincial patient-reported outcomes registry in Ontario that not only will facilitate more detailed evaluation of the impact of attendance at chronic pain clinics on health care utilization but will yield standardized data based upon the current best practices for clinical outcome measurement that will provide important data to (1) inform policy development, (2) stimulate pediatric pain research, (3) accelerate our understanding of the impact of pediatric chronic pain on society, and (4) enhance the effectiveness of care that we can provide. In conclusion, our retrospective analysis demonstrated a reduction in health care cost and utilization. Further prospective research is required to establish whether attendance at the chronic pain clinic caused this reduction in health care costs and, if this is found to be so, to identify the effective components of treatment. Further research into patient outcomes and impact on health care utilization is urgently required and will be well served by the development of a pediatric chronic pain registry.

## Disclosure of Interest

Garry Salisbury declares no conflicts of interest and has received no perquisite from any source related to this work. Vitali Ostapets has no conflict of interest to declare. Jennifer Stinson has no conflict of interest to declare. Carley Ouellette has no conflict of interest to declare. Fiona Campbell has no conflict of interest to declare.
